# Compared to non-drinkers, individuals who drink alcohol have a more favorable multisystem physiologic risk score as measured by allostatic load

**DOI:** 10.1371/journal.pone.0223168

**Published:** 2019-09-30

**Authors:** Deena Goldwater, Arun Karlamangla, Sharon Stein Merkin, Teresa Seeman

**Affiliations:** Department of Medicine, Division of Geriatrics, David Geffen School of Medicine at UCLA, Los Angeles, California, United States of America; Johns Hopkins University Bloomberg School of Public Health, UNITED STATES

## Abstract

**Aims:**

Alcohol use is associated with both positive and negative effects on individual cardiovascular risk factors, depending upon which risk factor is assessed. The present analysis uses a summative multisystem index of biologic risk, known as allostatic load (AL), to evaluate whether the overall balance of alcohol-associated positive and negative cardiovascular risk factors may be favorable or unfavorable.

**Methods:**

This analysis included 1255 adults from the Midlife in the United States (MIDUS) biomarker substudy. Participants, average age 54.5 (±11) years, were divided into 6 alcohol-use categories based on self-reported drinking habits. Current non-drinkers were classified as lifelong abstainers and former light drinkers, former moderate drinkers, or former heavy drinkers. Current alcohol users were classified as light, moderate, or heavy drinkers. A total AL score was calculated using 24 biomarkers grouped into 7 physiologic systems including cardiovascular, inflammation, glucose metabolism, lipid metabolism, sympathetic and parasympathetic nervous systems, and the hypothalamic-pituitary-adrenal axis. Mixed-effects regression models were fit to determine the relationship between alcohol use categories and AL with controls for covariates that may influence the relationship between alcohol use and AL.

**Results:**

468 (37.6%) individuals were current non-drinkers while 776 (62.4%) were current drinkers. In adjusted mixed-effects regression models, all 3 groups of current drinkers had significantly lower average AL scores than the lifelong abstainer/former light drinker group (light: -0.23, 95% CI -0.40, -0.07, p < 0.01; moderate: -0.20, 95% CI -0.38, -0.02, p < 0.05; heavy: -0.30, 95% CI -0.57, -0.04, p < 0.05), while the average AL scores of former moderate and former heavy drinkers did not differ from the lifelong abstainer/former light drinker group.

**Conclusions:**

Current alcohol use is associated cross-sectionally with a favorable multisystem physiologic score known to be associated with better long-term health outcomes, providing evidence in support of long-term health benefits related to alcohol consumption.

## Introduction

Epidemiologic studies show an association between light to moderate alcohol consumption and reduced risk for adverse cardiovascular (CV) events [1–6]. When defined as ≤ 1 drink per day for women and ≤ 2 drinks per day for men, light to moderate drinkers experience fewer adverse CV events compared to both non-drinkers and heavy drinkers, with meta-analyses demonstrating up to a 25% reduction in both incident CV disease as well as CV mortality [2]. However, data re-analysis has raised questions regarding the validity of these findings, suggesting that many studies lack appropriate adjustment for confounding factors (i.e. socioeconomic status, chronic disease, social support, etc.) or use suboptimal statistical methodology [7–9]. Indeed, a recent meta-analysis accounting for these issues was unable to confirm a favorable relationship between alcohol use and survival [9].

Although the relationship to long-term outcomes remains debated, on a physiologic level, alcohol does appear to modulate cardiovascular risk factors. In a dose-dependent manner, ethanol, the primary active ingredient in alcoholic beverages, has potentially cardioprotective as well as toxic effects on biomarkers of cardiovascular risk, depending upon which biomarker is being evaluated. Indeed, randomized trials have demonstrated that alcohol consumption beneficially affects known biomarkers of cardiovascular disease including lipid, inflammatory, and insulin-sensitivity profiles by increasing levels of high-density lipoprotein cholesterol (HDL-C) [10], decreasing levels of fibrinogen and C-reactive protein [11], and improving insulin sensitivity [12,13].

In contrast, alcohol has also been shown to have less beneficial effects on other markers of cardiovascular risk. In addition to elevated blood pressure [14], dose-dependent alcohol consumption unfavorably alters the activity of hypothalamic-pituitary-adrenal (HPA) axis. The resulting abnormalities include increased baseline cortisol levels, blunted diurnal cortisol rhythm [15], increased circulating catecholamines [16], and diminished stress-response inhibition [17], all of which have been associated with incident CV disease and adverse events. Moreover, the HPA axis changes persist in heavy drinkers even after alcohol abstinence [18].

The relative balance of the physiologic benefits of alcohol use versus the harms remains unclear. Clarifying changes across multiple physiologic systems simultaneously may provide more insight into the mechanisms by which alcohol use may confer cardiovascular protection versus toxicity. Allostatic load (AL), a multisystem index of biological risk, has been operationalized as a summative measure of physiologic dysregulation across major regulatory systems including cardiovascular, autonomic, metabolic, neuro-endocrine, and immune systems [19]. Higher levels of AL have been associated with cardiovascular disease and all-cause mortality [20–23], while decreases in AL over time are associated with improved survival [22].

The relationship between alcohol use and AL is incompletely explored. One study investigating the effects of multiple health behaviors on AL found that both current alcohol use (dichotomized into current drinkers vs never/former drinkers) was associated with lower AL [24]. When included as a binary covariate (current drinkers vs never/former drinkers) in models exploring the relationship between AL and neighborhood disadvantage, current alcohol use was again shown to be associated with lower AL [25,26]. However, to our knowledge, there are no dedicated analyses exploring the relationship between alcohol use and AL that address the epidemiologic data suggesting that light to moderate alcohol consumption may have more cardiovascular benefit compared to either abstinence or heavy drinking.

To continue to address the ongoing debate on the relationship between alcohol consumption and long-term cardiovascular health benefits, the objective of this study was to clarify the association between alcohol consumption and AL. We hypothesized that individuals who drink light to moderate amounts of alcohol would demonstrate lower AL than both non-drinkers and heavy drinkers. Using cross-sectional data from the Biomarker Project of the second wave of the Midlife in the United States (MIDUS) study, we examined whether self-reported alcohol use was a predictor of AL. We also performed preliminary explorations into the relationship between the 7 AL physiologic systems (i.e. cardiovascular, glucose metabolism, lipid metabolism, inflammation, sympathetic and parasympathetic nervous systems, and hypothalamic-pituitary-adrenal axis) and alcohol use.

## Materials and methods

The cross-sectional data for this study were obtained from a longitudinal study of health and aging in the United States called Midlife in the United States (MIDUS) study. Extensive information on MIDUS recruitment and survey protocols have been previously published [27]. Briefly, between 1994–1995, the first wave of 7,108 MIDUS participants (MIDUS 1) were recruited by random telephone digit dialing and completed a comprehensive survey via telephone. Questions focused on a wide range of psychological, social, behavioral, and health-related factors. All participants were between the ages of 25–74 years old and English-speaking. After an average of 9 years (range = 7.8–10.4 years), the original MIDUS cohort was resurveyed by telephone (MIDUS 2). After adjusting for mortality, longitudinal follow up rate was 75%. During MIDUS 2, an additional cohort of black Americans was recruited from Milwaukee, WI, known as MIDUS Milwaukee (n = 592). In 2005, individuals recruited for MIDUS Milwaukee were age 35–85 years old and participated in surveys that paralleled those of the main MIDUS 2 cohort.

The cross-sectional data utilized herein were obtained during the second wave of MIDUS from a subset of individuals from MIDUS 2 (n = 1,054) and MIDUS Milwaukee (n = 201) who also participated in the Biomarker substudy [28]. With respect to biomarker collection, between July 2004 and May 2009, participants were assigned to present to one of three General Clinical Research Centers (University of California, Los Angeles, Georgetown University, or University of Wisconsin) for a 24-hour stay. The Biomarker study protocol consisted of a physical exam as well as biosample collection that included a fasting morning blood draw and 12-hour overnight urine collection. Additional details of this protocol have been published previously [28].

### Alcohol use

Individuals were asked a number of survey questions related to alcohol use via mail-in self-administered questionnaire (MIDUS 2 main sample) or in-person survey (MIDUS Milwaukee sample). Participants reported age the first time he or she had a drink, the number of drinks currently consumed per week (or, if less than one per week, number of drinks per month), and how much a person was drinking at the time in their life when they were drinking the most. The questions regarding *current* drinking habits included: “During the last month, how often did you drink any alcoholic beverages, on the average? Would you say every day, 5 or 6 days a week, 3 or 4 days a week, 1 or 2 days a week, or less often than 1 day a week?” Individuals answering “less often than 1 day a week” would be further questioned if they drank “3 to 4 days per month, 1 to 2 days per month, or less often than that?” Participants were told to count as one 'drink', a bottle of beer, a wine cooler, a glass of wine, a shot of liquor, or a mixed drink. Participants were then asked: “On the days when you drank, about how many drinks did you drink, on the average?” The number of drinks per week was calculated by multiplying the answer to these two survey questions. Of note, with respect to the first question, we used the lower value of the range (i.e. 3 was used if an individual answered that they drink “3 or 4 days a week.”) in the calculation. Therefore, the average number of drinks per week may be underestimated in some individuals. Participants were also asked about *current* binge drinking habits with the question, “Considering all types of alcoholic beverages, how many times during the past month did you have 5 or more drinks on the same occasion?” Similar questions related to *past* alcohol use were also asked, including: “At the time you drank most, would you say you drank every day, 5 or 6 days per week, 2 or 3 days per week, 1 day per week, or less than once per week?” Similar to current drinking questions, individuals who stated they drank less than once per week in the past were further questioned if they drank “3 to 4 days per month, 1 to 2 days per month, or less often than that?”

Participants were categorized into 6 alcohol-related groups: 1) Lifelong abstainers and former light drinkers, 2) former moderate drinkers, 3) former heavy drinkers, 4) current light drinkers, 5) current moderate drinkers, and 6) current heavy drinkers. Individuals answering, “never had a drink” to the question, “How old were you when you had your first drink?” were classified as lifelong abstainers. Individuals were considered to be “former” drinkers if they gave an age for when they had their first drink but answered “no” to the question, “During the past month, have you had at least one drink of any alcohol beverage, such as beer, wine, wine coolers, or liquor?” Light drinking was considered to be having ≥ 1 drink per month but < 1 drink per week; moderate drinking was considered to be having ≥ 1 drink per week but < 8 drinks per week for women and < 15 drinks per week for men; heavy drinking was considered to be ≥ 8 drinks per week for women or ≥ 15 drinks per week for men [29]. Of note, data suggests that up to 50% of individuals reporting to be lifelong abstainers change their answer upon repeat questioning. Therefore, based on previously published data and recommendations, irregular lifetime drinkers (i.e. former light drinkers) were combined with lifelong abstainers as the reference category [7]. An additional alcohol variable was created for current drinkers: Individuals were classified as binge drinkers if they reported that they had ≥ 5 drinks on any one occasion in the last month [29]. Gender-specific binge drinking criteria (i.e. ≥ 4 drinks on one occasion for women and ≥ 5 drinks for men) were not utilized, as the MIDUS survey questions asked only about ≥ 5 drinks on one occasion regardless of gender. Individuals were excluded if they did not know or refused to answer how many days per week or month they drink, how many drinks on average they drink on one occasion, and if they refused to answer or did not know if they drank more than 5 drinks on any one occasion in the last month (n = 11).

### Allostatic load

Consistent with previous work, the AL index is designed as a summative measure to capture dysregulation across multiple physiological systems [30–32]. System indicators and biomarker components of AL were selected based on the fact that they are either a known marker of physiologic system function or a known marker of biological risk, as well as feasibly collectible within the logistical and financial constraints of the MIDUS study. As done previously, biomarkers were organized into 7 physiologic subscales (Table 1) [30,32]. The 1) *cardiovascular* subscale included systolic blood pressure (SBP), pulse pressure, and resting pulse rate; the 2) *sympathetic nervous system* measurements included 12-hour overnight urine epinephrine and norepinephrine concentrations; the 3) *parasympathetic nervous system* activity was measured with respect to heart rate variability indicators, including low frequency spectral power, high frequency spectral power, the standard deviation of R-R (heartbeat to heartbeat) intervals, and the root mean square of successive differences; the 4) *hypothalamic-pituitary-adrenal (HPA) axis* activity was measured by 12-hour overnight urine cortisol concentrations as well as serum levels of the hormone dehydroepiandrosterone sulfate (DHEAS); the 5) *inflammation* subscale included plasma C-reactive protein (CRP), fibrinogen, and serum measures of interleukin-6 (IL6), E-selectin, and intracellular adhesion molecule-1 (ICAM-1); the 6) *lipid metabolism* subscale incorporated concentrations of HDL cholesterol, low density lipoprotein (LDL) cholesterol, and triglycerides, as well as body mass index (BMI) and waist-hip ratio; and finally, the 7) *glucose metabolism* subscale included measures of glycosylated hemoglobin, fasting glucose, and the homeostatic model assessment for insulin resistance (HOMA-IR).

**Table 1:** Allostatic load biomarkers by physiologic subscale.

Physiologic subscale	Biomarkers	Quartile-based cut points for allostatic load scoring
Cardiovascular	Systolic blood pressure (mmHg)*	≥ 143
	Pulse pressure (mmHg)	≥ 65
	Resting pulse rate (beats per minute)*	≥ 77
Sympathetic nervous system	12h overnight urine epinephrine (mg/g of creatinine)	≥ 2.54
	12h overnight urine norepinephrine (mg/g of creatinine)	≥ 33.3
Parasympathetic nervous system	Heart rate variability indicators:	
	-low frequency spectral power (msec^2^)	≤ 114
	-high frequency spectral power (msec^2^)	≤ 54.2
	-standard deviation of R-R interval (msec)	≤ 23.5
	-root mean square of successive differences in R-R variability (msec)	≤ 11.8
Hypothalamic-pituitary-adrenal axis	12h overnight urine cortisol (mg/g of creatinine)	≥ 21.0
	Serum dehydroepiandrosterone sulfate (DHEAS)	≤ 51.0
Inflammation	C-reactive protein (mg/L)	≥ 3.18
	Fibrinogen (mg/dL)	≥ 390
	Interleukin-6 (ng/L)	≥ 3.18
	E-selectin (ng/mL)	≥ 50.6
	Intracellular adhesion molecule-1 (mg/L)	≥ 330
Lipid metabolism	Body mass index (kg/m^2^)	≥ 32.3
	Waist-hip ratio	≥ 0.97
	High density lipoprotein cholesterol (mg/dL)	≤ 41.4
	Low density lipoprotein cholesterol (mg/dL)*	≥ 128
	Triglycerides (mg/dL)*	≥ 160
Glucose metabolism	Glycosylated hemoglobin (%)*	≥ 6.1
	Fasting glucose (mg/dL)*	≥ 105
	Homeostatic model assessment for insulin resistance	≥ 4.04

*Scored as high-risk if taking medications designed to impact these risk factors, even if biomarker value is below the cut-point.

The total AL score is a summative measure of the 7 subscale scores. To calculate each subscale, first, each system indicator or biomarker was divided into quartiles. Then, either the lowest or highest quartile for each indicator or biomarker was classified as high risk, depending on whether low or high values traditionally indicate worse health status or outcomes. The cut point values for high risk for each AL biomarker in this cohort have been published previously and are shown again in Table 1 [32]. Subscale scores are continuous, from 0–1, depending upon the proportion of the subscale indicators that fell into the high-risk categories. Therefore, the total AL score is a continuous measure, ranging from 0–7, with higher scores reflecting greater dysregulation across systems. Of note, use of medications is included for individual biomarkers. Specifically, use of 1) antihypertensive medications would be reflected as a high risk score for SBP; 2) anti-nodal agents would be reflected as high-risk score for resting pulse; 3) diabetes medications would be reflected as a high-risk score for fasting glucose and glycosylated hemoglobin; 4) statins would be reflected as a high-risk score for LDL, and 5) fibrates would be reflected as high-risk for triglycerides [31]. With the exception of the parasympathetic system, for which 8% of respondents were missing data due to technical or instrument difficulties, rates of missing were very low. Subscale scores were calculated if at least half of the biomarkers assigned to the system were measured. Fewer than 20 participants received system scores based on incomplete biomarker data. Total AL scores were only computed if at least 6 of 7 subscale scores were available. For participants missing only the parasympathetic subscale, we imputed AL score from participants’ scores on the other six systems, age, gender, and race/ethnicity, using a regression equation derived from those with complete biomarker data. For four participants who were each missing exactly one of the other six system scores, the missing system score was imputed as zero (because the sample median for four of the seven system scores was zero). A flag was created for individuals with an imputed parasympathetic subsystem score, which was included as a covariate in subsequent regression analyses. The vast majority of participants (90.5%) had data for all 7 systems.

### Covariates

Covariates were selected based on best available data indicating that they might be confounding factors in the relationship between alcohol use and the AL score. Sociodemographic covariates included age, gender, race (white/non-white), and a measure of socioeconomic status (SES). Briefly, the SES score was a composite measure of education, current income, childhood family income, availability of money to meet needs, and ability to pay bills. The SES score (0–10) is the sum of each component variable, which were coded from 0–2 [30]. Health behaviors such as smoking status (current, former, never), and > 20 min of physical activity 3 or more days per week (yes/no) were also included, as was self-reported health (continuous variable from 0–10). Finally, the self-reported history of major chronic diseases were included, namely: heart disease, transient ischemic attack (TIA) /stroke, cancer, cirrhosis, peptic ulcer disease (PUD), and chronic obstructive pulmonary disease (COPD)/emphysema. Neither hypertension nor diabetes was included as a covariate, as indicators of both are specific elements of the AL score.

### Analysis

Mixed-effects regressions were fit to determine the association between 6 alcohol consumption patterns (lifelong abstainer and former light, former moderate, former heavy, current light, current moderate, and current heavy) and AL. Given that siblings were recruited for MIDUS and comprised 16% of the total Biomarker cohort, mixed-effects models were utilized to control for clustering within families. Models were fully adjusted for the covariates listed above, as well as a quadratic term for age (age^2^), and the following interactions: age x gender, race x SES, self-reported health x SES, and gender x SES. Additionally, given that binge-drinking behavior likely has health-consequences beyond total number of drinks consumed [33], we also included a control for binge-drinking (yes/no). Given that the MIDUS study was not developed to be a nationally representative sample, nor does it claim to be so, survey weights are not applicable and were not utilized in this analysis.

To address the possibility that individuals who are “former” drinkers quit drinking due to health-related issues (i.e. the “sick quitter” hypothesis [8,9]), and therefore would have higher AL scores, a sensitivity analysis was performed in a “healthy” cohort in which all individuals with a self-reported major chronic disease (i.e. heart disease (n = 144, 11.5%), TIA/stroke (n = 54, 4.3%) cancer (n = 170, 13.6%), cirrhosis (n = 28, 2.2%), PUD (n = 67, 5.4%), and COPD/emphysema (n = 36, 2.9%)) were removed from the analyses, leaving a total sensitivity analysis cohort of 866 individuals. Using this group, the same mixed-effects regression models described above were fit to determine the association between alcohol consumption patterns and AL. Furthermore, this cohort was used for additional exploratory analyses to assess the relationship between individual AL subscales and alcohol consumption patterns.

Finally, using fully adjusted mixed-effects regression models, additional exploratory analyses were performed with both the entire cohort as well as the “healthy” cohort to test for modification by gender and race on the AL-alcohol use association.

## Results

Baseline characteristics and summary statistics are presented by drinking category for the 1255 individuals in the MIDUS biomarker project in Table 2. The average age of the cohort was 54.5 (±11) years old, 56.8% were men, and 22.8% were non-white. With respect to drinking categories, 468 (37.6%) individuals were current non-drinkers, while 776 (62.4%) participants reported having had at least one drink in the last month, with 11 (0.9%) individuals missing data on alcohol consumption. Of the non-drinkers, 284 (60.6%), 109 (23.3%), and 75 (16%) were lifelong abstainers/former light drinkers, former moderate drinkers, and former heavy drinkers, respectively. Of the current drinkers, 357 (46%), 350 (45%), and 69 (8.9%) were light, moderate, and heavy drinkers, respectively. Additionally, 166 (21.4%) of current drinkers reported at least one incidence of binge drinking, including 9.5%, 25.1%, and 63.7% of light, moderate, and heavy drinkers, respectively. The average AL score for the entire group was 1.97 (±1.15). The average AL score for the physiologic subscales, as well as a breakdown of AL scores by drinking category, can be found in Table 2. The average values for individual AL indicators and biomarkers are available in the Table A in the S1 File.

**Table 2:** Baseline characteristics of the MIDUS biomarker cohort by alcohol use category.

	Total (n = 1244)	Abstainer/ Former light (n = 284)	Former moderate (n = 109)	Former heavy (n = 75)	Current light (n = 357)	Current moderate (n = 350)	Current heavy (n = 69)
**Age**	54.5 (±11.7)	57.0 (±12.0)	52.7 (±11.4)	53.9 (±10.6)	53.7 (±11.4)	54.7 (±12.0)	51.4 (±10.9)
**Gender**							
Female	542 (43.2)	195 (68.6)	63 (57.8)	30 (40.0)	236 (66.1)	147 (42.0)	38 (55)
**Non-white**	286 (22.8)	86 (30.2)	26 (23.8)	17 (22.7)	72 (20.2)	57 (16.3)	25 (36.2)
**SES**	5.2 (±2.8)	4.7 (±2.8)	4.4 (±2.6)	4.3 (±3.0)	5.5 (±2.7)	4.8 (±2.8)	4.6 (±3.3)
**Physical activity**							
30 min at least 3 x/week	960 (76.5)	214 (75.3)	79 (72.4)	53 (70.6)	279 (78.2)	274 (78.3)	52 (75.4)
**Self-rated health**	7.3 (±1.6)	7.3 (±1.9)	7.1 (±1.6)	7.3 (±1.6)	7.5 (±1.5)	7.7 (±1.3)	7.1 (±1.8)
**Smoking status**							
Current	187 (14.1)	31 (10.9)	16 (14.7)	20 (26.7)	42 (11.8)	52 (14.9)	25 (36.2)
Former	409 (32.6)	51 (18.0)	45 (41.3)	40 (53.3)	105 (29.4)	137 (39.3)	26 (37.7)
Never	658 (52.5)	202 (71.1)	48 (44.0)	15 (20.0)	210 (58.8)	160 (45.8)	18 (26.1)
**Drinks per week**							
Current (n = 776)	4.4 (9.1)	N/A	N/A	N/A	0.8 (0.5)	4.6 (3.5)	22.8 (21.4)
Former (n = 363)*	11.0 (27.2)	1 (0.8)	4.9 (3.3)	43.9 (47.2)	N/A	N/A	N/A
**Current binge drinker**	166 (13.2)	N/A	N/A	N/A	34 (9.5)	88 (25.1)	44 (63.8)
**Past medical history:**							
Heart disease	141 (11.4)	40 (14.1)	11 (10.2)	11 (14.7)	29 (8.2)	45 (12.9)	5 (7.2)
TIA/Stroke	54 (4.4)	19 (6.6)	3 (2.8)	3 (4.0)	10 (2.8)	14 (4.0)	5 (7.2)
Cancer	169 (13.6)	40 (3.2)	9 (8.3)	10 (13.3)	60 (16.9)	43 (12.2)	7 (10.1)
PUD	64 (5.2)	17 (6.0)	7 (6.6)	4 (5.3)	19 (5.3)	13 (3.8)	4 (5.9)
Cirrhosis	28 (2.3)	5 (2.8)	3 (2.3)	5 (6.7)	7 (2.0)	6 (1.7)	2 (2.9)
COPD	36 (2.9)	11 (3.8)	2 (1.8)	3 (4.0)	9 (2.5)	9 (2.6)	2 (2.9)
**Allostatic load total**	1.96 (±1.2)	2.3 (±1.2)	2.1 (±1.1)	2.1 (±1.1)	1.8 (±1.1)	1.8 (±1.1)	1.8 (±1.0)
**Allostatic load subscales**							
Cardiovascular	0.38 (±0.34)	0.44 (±0.36)	0.40 (±0.34)	0.39 (±0.33)	0.37 (±0.33)	0.35 (±0.34)	0.39 (±0.31)
HPA axis	0.24 (±0.31)	0.29 (±0.32)	0.22 (±0.28)	0.25 (±0.32)	0.23 (±0.30)	0.22 (±0.31)	0.19 (±0.27)
Inflammation	0.28 (±0.28)	0.31 (±0.27)	0.30 (±0.29)	0.33 (±0.29)	0.27 (±0.27)	0.23 (±0.25)	0.27 (±0.25)
Glucose metabolism	0.29 (±0.35)	0.34 (±0.39)	0.39 (±0.39)	0.27 (±0.38)	0.27 (±0.33)	0.26 (±0.32)	0.21 (±0.30)
Lipid metabolism	0.30 (±0.26)	0.31 (±0.25)	0.34 (±0.29)	0.36 (±0.27)	0.28 (±0.25)	0.30 (±0.27)	0.28 (±0.26)
Parasympathetic	0.24 (±0.36)	0.28 (±0.38)	0.20 (±0.34)	0.29 (±0.39)	0.22 (±0.35)	0.23 (±0.35)	0.20 (±0.31)
Sympathetic	0.23 (±0.35)	0.29 (±0.38)	0.23 (±0.34)	0.24 (±0.32)	0.21 (±0.33)	0.23 (±0.35)	0.21 (±0.34)

Data presented as n (% of column total) or mean (±SD)

*Does not include the lifelong abstainers (n = 107)

**MIDUS**- Midlife in the United States

**SES**-Socioeconomic status: On a scale of 0–10, with 10 being the most optimal status. Composite measure of education, current income, childhood family income, availability of money to meet needs, and ability to pay bills.

**Self-rated health**: On a scale of 0–10, with 10 being the healthiest

**Current binge drinker:** Reported at least 1 episode of ≥ 5 drinks on one occasion

**Past medical history:** Self-reported history of disease. TIA-transient ischemic attack; PUD-Peptic ulcer disease; COPD-Chronic Obstructive Pulmonary Disease

**Allostatic load subscales:** HPA- hypothalamic-pituitary-adrenal

In unadjusted mixed-effects regression models, we found that all 3 groups of current drinkers had significantly lower AL scores than non-drinkers (Table 3). Compared to lifelong abstainer/former light drinkers, light, moderate, and heavy drinkers had significantly lower AL scores (light: -0.39, 95% CI -0.57, -0.22, p < 0.0001; moderate: -0.39, 95% CI -0.58, -0.21, p < 0.0001; heavy: -0.47, 95% CI -0.76, -0.19, p = 0.001), while the AL scores of former moderate and former heavy drinkers did not differ from the lifelong abstainer/former light drinker group These results were confirmed in fully adjusted mixed-effects regression models in which we similarly found that all 3 groups of current drinkers had significantly lower AL scores than current non-drinkers (Table 3). Specifically, light, moderate, and heavy drinkers had significantly lower AL scores than lifelong abstainer/former light drinkers (light: -0.23, 95% CI -0.40, -0.07, p < 0.01; moderate: -0.20, 95% CI -0.38, -0.02, p < 0.05; heavy: -0.30, 95% CI -0.57, -0.04, p < 0.05), while the AL scores of former moderate and former heavy drinkers did not differ from the lifelong abstainer/former light drinker group (Table 3).Of note, consistent with previous work, better self-reported health, more physical activity, and higher SES were also associated with lower AL scores, -0.14 (p < 0.0001), -0.21 (p < 0.01), and -0.03 (p < 0.05), respectively (data not tabulated). The binge drinking flag was not associated with AL.

**Table 3:** Association of allostatic load with drinking categories.

	β (95% CI)	P	β (95% CI)	P
**Full MIDUS biomarker cohort** **(N = 1194)**	**Unadjusted model**		**Adjusted**^**†**^ **model**	
Lifelong abstainer / former light	reference		reference	
Former moderate	-0.19 (-0.43, 0.05)	0.52	-0.08 (-0.31, 0.16)	0.53
Former heavy	-0.19 (-0.47, 0.09)	0.40	-0.11 (-0.38, 0.15)	0.41
Current light	-0.39 (-0.57, -0.22)	< 0.0001	-0.23 (-0.40, -0.07)	0.006
Current moderate	-0.39 (-0.58, -0.21)	< 0.0001	-0.20 (-0.38, -0.02)	0.03
Current heavy	-0.47 (-0.76, -0.19)	0.001	-0.30 (-0.57, -0.04)	0.02
Sensitivity analysis in healthy cohort** (N = 866)	**Unadjusted model**		Adjusted* **model**	
Lifelong abstainer / former light	reference		reference	
Former moderate	-0.28 (-0.58, 0.01)	0.06	-0.15 (-0.43, 0.14)	0.30
Former heavy	-0.05 (-0.41, 0.32)	0.80	0.08 (-0.27, 0.43)	0.65
Current light	-0.40 (-0.62, -0.19)	< 0.001	-0.28 (-0.47, -0.08)	0.006
Current moderate	-0.40 (-0.63, -0.17)	< 0.001	-0.22 (-0.44, -0.005)	0.04
Current heavy	-0.50 (-0.84, -0.06)	0.003	-0.35 (-0.66, -0.05)	0.02

*Adjusted for: age, gender, race, SES, physical activity, smoking status, self-reported health, binge drinking, age^2^, age x gender, race x gender, race x SES, gender x SES, self-reported health x SES, as well as past medical history of heart disease, COPD, peptic ulcer disease, cirrhosis, cancer, and TIA/stroke.

**Sensitivity analysis cohort excluded all individuals with a self-reported history of the following major chronic diseases: heart disease, cancer, peptic ulcer disease, cirrhosis, and chronic obstructive pulmonary disease.

^**†**^Sensitivity analyses were adjusted for all covariates listed above except heart disease, cancer, peptic ulcer disease, cirrhosis, TIA/stroke and COPD.

SES–socioeconomic status; COPD–chronic obstructive pulmonary disease; TIA–transient ischemic attack

Post-hoc analyses were performed to assess for a dose-response with respect to alcohol use and AL. Both unadjusted and fully adjusted mixed-effects regression models were fit using the 3 groups of current drinkers (light, moderate, and heavy) as the predictors of AL with current light drinkers as the reference group. In the total MIDUS cohort of current drinkers, no significant differences were found between groups in either the unadjusted (n = 774; moderate: 0.01, p = 0.94; heavy: -0.05, p = 0.72) or fully adjusted models (n = 751; moderate: 0.21, p = 0.79; heavy: -0.8, p = 0.52) (data not tabulated).

To address the “sick-quitter” hypothesis, in which individuals who have quit drinking are thought to have done so for health reasons and are therefore “sicker” than current drinkers, sensitivity analyses were performed on a cohort in which individuals with a self-reported medical history of heart disease, TIA/stroke, cancer, cirrhosis, PUD, and COPD/emphysema were removed (n = 866). In both unadjusted and fully adjusted mixed-effects regression models in this sensitivity analysis, we confirmed that all groups of current drinkers had lower AL scores compared to non-drinkers (Table 3). In the unadjusted models, AL was lower in current light, moderate, and heavy drinkers compared to the reference group (light: -0.40, 95% CI -0.62, -0.19, p < 0.001; moderate: -0.40, 95% CI -0.63, -0.17, p < 0.05; heavy: -0.50, 95% CI -0.84, -0.06, p < 0.05), with similar results in the fully adjusted models (light: -0.28, 95% CI -0.47, -0.09, p < 0.01; moderate: -0.22, 95% CI -0.44, -0.01, p < 0.05; heavy: -0.36, 95% CI -0.66, -0.06, p < 0.05). In neither model were AL scores of former moderate and former heavy drinkers significantly different than the lifelong abstainers/former light drinkers group (Table 3). Of note, better self-reported health and more physical activity remained associated with a lower AL score, -0.16 (p < 0.0001) and -0.23 (p< 0.01), respectively, while a higher SES did not (data not tabulated). Again, binge drinking was not significantly associated with the AL score.

To understand the relationship between the individual AL subscales and alcohol use, exploratory analyses were performed in the “healthy” cohort to assess the relationship between drinking status and individual subscales. Fully adjusted mixed-effects regression models showed that only two of the individual AL subscales were significantly related to drinking status. Compared to the lifelong abstainer/former light drinker group, the sympathetic nervous system score was significantly lower only in current light drinkers (-0.09, 95% CI -0.13, -0.02, p < 0.05), while the glucose metabolism score was significantly lower only in current heavy drinkers (-0.18, 95% CI -0.30, -0.07, p < 0.01). The cardiovascular, inflammation, parasympathetic, lipid metabolism, and HPA axis subscales did not show a significant association with drinking status (Table B in S1 File).

Additional sensitivity analyses were performed to investigate modification by gender and race on the alcohol-AL association. The interaction between gender and alcohol use categories was non-significant in both the total cohort as well as the “healthy” cohort (p = 0.54 and p = 0.55, respectively). The interaction between race and alcohol use categories was borderline significant in the total cohort (p = 0.052) and reached significance in the “healthy” cohort (p < 0.05) (data not tabulated). Post-hoc analyses stratified by race were therefore performed. Utilizing the total MIDUS biomarker cohort, fully adjusted models demonstrated that in white individuals (n = 923), light (-0.33, 95% CI -0.51, -0.14, p < 0.001), moderate (-0.33, 95% CI -0.52, -0.14, p < 0.01), and heavy (-0.48, 95% CI -0.82, -0.13, p < 0.001) drinking was associated with a significantly lower AL than the reference group of abstainers/former light drinkers. This relationship held true in the white “healthy” cohort (n = 646) where light (-0.39, 95% CI -0.60, -0.18, p < 0.01), moderate (-0.43, 95% CI -0.65, -0.20, p < 0.01), and heavy (-0.59, 95% CI -1.02, -0.16, p < 0.01) drinking were associated with lower AL than the reference group. In the total MIDUS cohort of non-white individuals (n = 271), compared to the reference group of abstainers/former light drinkers, no significant relationship was found between light (0.01, p = 0.97), moderate (0.07, p = 0.74), or heavy (0.11, p = 0.73) drinking and AL. These findings also held true in the “healthy” non-white cohort (n = 200), where light (0.02, p = 0.92), moderate (0.25, p = 0.29), and heavy (0.14, p = 0.68) drinking were not associated with AL (Table C in the S1 File).

## Discussion

Using a national, longitudinal study of health and well-being of middle-age men and women in the United States, we set out to explore the relationship between self-reported alcohol intake and a measure of multisystem physiologic dysfunction, allostatic load. We found that individuals who reported drinking any amount of alcohol in the last month had lower AL than non-drinkers. Specifically, we found that each category of current drinkers, including current light, regular, and heavy drinkers, had significantly lower AL than the reference group of individuals who reported either lifelong alcohol abstinence or former light drinking. To address the “sick-quitter” hypothesis, a sensitivity analysis performed with a cohort of participants without a self-reported history of chronic disease confirmed the findings that current light, regular, and heavy drinkers all had lower AL scores than the reference group of lifelong abstainers and former light drinkers.

Alcohol use has been shown to have both positive and negative effects on individual cardiovascular risk factors depending upon which risk factor is being considered, but whether the overall balance of alcohol-associated positive and negative effects is favorable or unfavorable has not been fully explored. Previous cross-sectional data has shown alcohol to be favorably associated with increased HDL levels [10], decreased markers of inflammation [11], and improved insulin sensitivity [12]. On the other hand, other cross-sectional studies have shown alcohol intake to be unfavorably associated with elevated blood pressure [16], heightened sympathetic nervous system activity as measured by increased catecholamine release [16], and dysregulated HPA axis activity [15]. By demonstrating an inverse relationship between current alcohol use and AL, the results presented here suggest that the net impact of current alcohol use may be one of cumulative multisystem physiologic benefit. Indeed, AL has been shown to strongly predict future adverse clinical outcomes such as cardiovascular events, cognitive impairment, and mortality [20–22]. However, future work to understand how alcohol-associated AL scores relate to long-term cardiovascular and non-cardiovascular (i.e. malignancy; liver failure) outcomes is necessary.

To further define the multisystem effects of alcohol use, we explored the relationship between individual AL subscales and drinking patterns. After excluding participants with self-reported history of major chronic medical issues, our preliminary results were remarkable in that only two subscales demonstrated a significant relationship with alcohol use. Namely, current light drinkers had more favorable sympathetic nervous system activity compared to nondrinkers, while heavy drinkers had more favorable metabolic glucose regulation (Table B in the S1 File). Although future studies exploring these specific relationships are required, these results suggest that the cross-sectional relationship between alcohol use and AL may be the result of the accumulation of small alcohol-related favorable physiologic changes across multiple physiological systems simultaneously, despite unfavorable effects on some individual system elements.

As mentioned above, the role of these noted alcohol-associated physiologic changes with respect to long-term outcomes remains unclear. The majority of epidemiologic data suggest a J-shaped curve for the relationship between alcohol intake and adverse health outcomes. Multiple large longitudinal studies have found self-reported light or moderate alcohol intake to be inversely associated with risk for cardiovascular disease related mortality and other major adverse cardiovascular events, while both heavy drinkers and those who abstain from alcohol have worse cardiovascular-related health outcomes and all-cause mortality [1–4,6,34]. However, controversy surrounding this relationship persists due to concerns with validity of self-reported alcohol use data [7], complexity of confounding factors [8,9], and various methodological statistical concerns [9]. Meta-analyses that have extensively adjusted for confounding factors and utilized more conservative statistical methods were unable to confirm the relationship between alcohol use and survival [8,9]. Although the relationship between alcohol use, AL, and long-term health outcomes is unknown, the cross-sectional analysis presented here, which was extensively adjusted for confounding factors and confirmed by a sensitivity analysis, demonstrates that alcohol use is associated with a favorable multisystem physiologic profile that is known to have a beneficial association with long-term health outcomes. The findings presented here support the possibility that alcohol use may indeed have some long-term health benefits.

It is important to note that our preliminary data suggest that race may play a role in the relationship between alcohol use and AL. While an inverse relationship between alcohol use and AL was present in whites, that relationship did not hold true in non-whites. It is possible that the lack of a relationship between AL and alcohol use in the non-white population is attributable to the small number of non-whites in the MIDUS Biomarker cohort. However, some data to suggest a more complex reason underlying this finding. Biomarkers utilized in the AL score may differ by race. For example, in white individuals, cardiovascular risk factors are associated with hypertriglyceridemia, whereas in black individuals, these same risk factors (i.e. cardiovascular disease, insulin resistance, and diabetes) are associated with normal triglyceride levels [35]. Additionally, there is some indication that the response of the neuroendocrine and immune systems to alcohol use may differ by race. Recent data suggests that for individuals who meet criteria for alcohol abuse (based on the Michigan Alcohol Screening Test), black individuals demonstrate higher urine norepinephrine [36], serum C-reactive protein, and serum interleukin-6 levels [37] compared to white individuals. These data warrant future studies exploring whether AL may need to be scored differently in race groups, as well as studies exploring the relationship between race, AL, and health behaviors. The Stress ancillary study of the Multi-ethnic study of atherosclerosis (MESA) [38], a cohort with racial/ethnic diversity as well as all the necessary AL biomarkers, is an ideal dataset with which to pursue this research.

To be consistent with the epidemiologic evidence that heavy alcohol users may be at higher risk for cardiovascular and all-cause mortality, we hypothesized that heavy alcohol use would be associated with an unfavorable AL profile. Our results do not support this hypothesis. Instead, we found that current heavy drinkers had significantly lower AL than non-drinkers. Moreover, post-hoc analyses evaluating for an alcohol dose-response did not show significant differences between current alcohol use groups. There are a few ways to reconcile this finding. First, it is notable that only 5.5% (n = 69) of participants met heavy drinking criteria. Therefore, future studies to confirm these findings in larger populations are indicated. Moreover, although there may be increased cardiovascular disease risk with heavy alcohol use, heavy drinkers are also at risk for other short-term (i.e. accidents, trauma, violence, risky behavior) and long-term health consequences (i.e. gastrointestinal cancers, cirrhosis, dementia) related to alcohol [4]. If the relationship between heavy drinking and favorable AL profile is confirmed in larger studies, it might suggest that the increased risk of mortality and disease are dependent to a greater extent on the effect of alcohol on specific tissues (i.e. cirrhosis, gastrointestinal tract malignancies) or short-term consequences such as accidents or trauma, than cardiovascular disease related deaths. Unfortunately, the limited number of mortalities in the current MIDUS biomarker cohort precludes mortality-specific analyses at this time.

This study has a number of potential limitations. The first is that alcohol use relied on self-report. Studies have shown a significant lack of consistency in self-reported alcohol intake over time. Furthermore, we were only able to determine whether a participant was a former drinker based on the answer to the question: “During the past month, have you had at least one drink of any alcohol beverage, such as beer, wine, wine coolers, or liquor?” Therefore, former drinkers had abstained from alcohol for at least one month, but it was not possible to determine the length of abstinence more specifically. An additional limitation is the lack of diversity in the MIDUS cohort (22.8% non-white), complicated by the fact that over 70% of the non-white participants were from the MIDUS Milwaukee cohort. These factors were adjusted for in the regression analyses, but exploring AL and alcohol use in cohorts with greater diversity is certainly warranted. Finally, as mentioned above, limited mortality to date in the MIDUS biomarker cohort unfortunately precludes attempts to investigate the relationship between alcohol use, AL, and long-term health outcomes at this time, although future work to address these questions is planned.

In conclusion, using a measure of multisystem physiologic function to quantify the balance of isolated positive and negative effects of alcohol on cardiovascular risk factors, we show that current alcohol users, compared to current non-drinkers, have significantly lower AL. Although the relationship between alcohol use, AL, and mortality has yet to be investigated, the results provide additional physiologic evidence for the decreased cardiovascular risk and improved long-term survival associated with responsible alcohol consumption noted in many epidemiologic studies.

## Supporting information

S1 File Supporting information. Table A: Baseline Biomarkers by alcohol use category; Table B: Association of AL subscales with drinking categories; Table C: Adjusted* association of AL with drinking categories by race.
(DOCX)
