# Supplementary material for: Compared to non-drinkers, individuals who drink alcohol have a more favorable multisystem physiologic risk score as measured by allostatic load
Source: PLoS One. 2019 Sep 30;14(9):e0223168. doi: 10.1371/journal.pone.0223168 (PMC6768542; doi:10.1371/journal.pone.0223168)
Supplement: S1 File — Table A: Baseline Biomarkers by alcohol use category; Table B: Association of AL subscales with drinking categories; Table C: Adjusted* association of AL with drinking categories by race. (DOCX) [file pone.0223168.s001.docx]

| **S1 Table A. Baseline biomarkers by alcohol use category** | | | | | | | |
| --- | --- | --- | --- | --- | --- | --- | --- |
|  | **Total**  **(n = 1244)** | **Abstainer/**  **former light**  **(n = 286)** | **Former moderate**  **(n = 109)** | **Former heavy**  **(n = 75)** | **Current light**  **(n = 357)** | **Current moderate**  **(n = 350)** | **Current heavy**  **(n = 69)** |
| **BMI (kg/m^2^)** | 29.8 (6.6) | 30.8 (7.4) | 30.7 (6.4) | 30.1 (6.5) | 29.8 (15.0) | 28.8 (5.6) | 28.5 (6.9) |
| **SBP (mmHg)** | 131.5 (18.3) | 134.8 (19.4) | 130.4 (17.0) | 131.2 (18.6) | 130.2 (18.2) | 130.5 (18.1) | 131.7 (15.0) |
| **DBP (mmHg)** | 75.7 (10.7) | 75.1 (10.6) | 75.0 (10.4) | 77.0 (9.7) | 74.8 (10.8) | 76.5 (11.0) | 78.4 (9.5) |
| **Pulse (bpm)** | 71.1 (11.2) | 72.2 (11.1) | 73.2 (11.8) | 70.7 (10.0) | 70.6 (11.1) | 70.1 (11.1) | 71.0 (11.8) |
| **Waist/hip ratio** | 0.9 (0.1) | 0.9 (0.1) | 0.9 (0.1) | 0.9 (0.1) | 0.9 (0.1) | 0.9 (0.1) | 0.9 (0.1) |
| **HDL (mg/dL)** | 55.4 (18.0) | 53.2 (16.8) | 51.7 (16.8) | 50.2 (17.4) | 55.5 (16.4) | 57.3 (19.9) | 64.3 (17.4) |
| **LDL (mg/dL)** | 105.5 (35.4) | 102.2 (34.4) | 108.6 (33.2) | 108.8 (32.5) | 109.4 (34.7) | 103.8 (37.4) | 102.6 (36.9) |
| **Triglycerides (mg/dL)** | 132.5 (131.8) | 133.3 (75.1) | 149.9 (204.1) | 129.3 (78.1) | 123.2 (71.7) | 134.4 (191.3) | 144.4 (90.4) |
| **Glucose (mg/dL)** | 102.1 (28.4) | 103.9 (26.7) | 105.3 (34.0) | 103.5 (31.4) | 99.7 (24.7) | 102.6 (32.8) | 98.6 (14.2) |
| **HA1c (%)** | 6.1 (1.2) | 6.3 (1.2) | 6.4 (1.5) | 6.1 (1.4) | 6.1 (1.1) | 6.0 (1.0) | 5.8 (0.6) |
| **HOMA-IR** | 3.6 (4.0) | 4.1 (4.7) | 4.2 (3.8) | 4.1 (5.5) | 3.4 (3.4) | 3.2 (3.8) | 3.0 (2.8) |
| **CRP (mg/L)** | 3.0 (4.8) | 2.8 (3.9) | 3.2 (4.4) | 2.8 (4.0) | 3.4 (5.7) | 2.8 (4.9) | 2.6 (3.8) |
| **IL6 (ng/L)** | 3.0 (3.0) | 3.2 (2.8) | 2.9 (2.2) | 2.9 (3.0) | 3.2 (3.6) | 2.8 (2.9) | 3.1 (2.8) |
| **Fibrinogen (mg/dL)** | 348.9 (87.8) | 358.4 (85.7) | 348.5 (79.0) | 344.7 (99.0) | 354.2 (87.0) | 341.3 (92.5) | 324.6 (69.1) |
| **E-selectin (ng/mL)** | 43.4 (22.7) | 45.6 (24.1) | 39.7 (20.2) | 48.4 (28.3) | 42.0 (22.0) | 42.0 (21.1) | 48.1 (24.6) |
| **ICAM-1 (mg/L)** | 288.5 (115.6) | 289.8 (116.3) | 289.6 (96.9) | 329.1 (141.5) | 281.2 (121.6) | 283.5 (105.5) | 289.1 (124.5) |
| **DHEAS (ug/dL)** | 116.5 (126.2) | 104.3 (126.3) | 126.6 (165.2) | 120.0 (131.1) | 115.4 (125.1) | 121.5 (115.1) | 131.8 (117.0) |
| **Urine Norepinephrine (mg/g creatinine)** | 27.4 (13.9) | 28.4 (13.6) | 28.7 (20.2) | 28.7 (14.9) | 27.3 (13.4) | 26.0 (12.0) | 27.6 (13.1) |
| **Urine Epinephrine (mg/g creatinine)** | 2.0 (1.3) | 2.1 (1.5) | 2.0 (1.3) | 2.0 (0.4) | 1.9 (1.2) | 1.9 (1.2) | 1.9 (1.0) |
| **Urine Cortisol (mg/g creatinine)** | 15.8 (24.6) | 18.6 (45.2) | 13.7 (11.2) | 13.2 (11.1) | 17.1 (17.0) | 14.2 (10.9) | 12.1 (9.2) |
| **HRV HF (ms^2^)** | 316.4 (776.9) | 300.0 (532.9) | 216 (282.2) | 242.9 (282.9) | 347.6 (1070.5) | 329.2 (798.8) | 402.2 (594.4) |
| **HRV LF (ms^2^)** | 409.1 (581.3) | 370.2 (592.2) | 341.8 (494.8) | 384.3 (444.4) | 400.9 (542.8) | 453.1 (630.1) | 527.5 (725.2) |
| Data presented as mean (SD)  **Biomarkers:** BMI – body mass index; SBP – systolic blood pressure; DBP – diastolic blood pressure; HDL – high density lipoprotein cholesterol; LDL – low density lipoprotein cholesterol; HA1c – glycosylated hemoglobin; HOMA-IR – homeostatic model assessment for insulin resistance; CRP – c-reactive protein; IL6 – interleukin 6; ICAM-1 – intracellular adhesion molecule 1; DHEAS – dihydroepiandrosterone sulfate; HRV HF – high frequency heart rate variability; HRV LF – low frequency heart rate variability | | | | | | | |

| **S1 Table B. Association of AL subscales with drinking categories** | | | | | | | |
| --- | --- | --- | --- | --- | --- | --- | --- |
|  | **Cardiovascular**  **(n = 851)** | **Sympathetic nervous system**  **(n = 842)** | **Parasympathetic nervous system**  **(n = 803)** | **Hypothalamic-pituitary-adrenal axis**  **(n=849)** | **Inflammation**  **(n = 844)** | **Lipid metabolism**  **(n = 848)** | **Glucose metabolism**  **(n = 841)** |
| **Abstainer/**  **Former light** | reference | reference | reference | reference | reference | reference | reference |
| **Former moderate** | -0.04  (-0.12, 0.05) | -0.06  (-0.15, 0.02) | -0.06  (-0.15, 0.04) | -0.02  (-0.10, 0.05) | -0.03  (-0.10, 0.04) | 0.04  (-0.04, 0.11) | 0.06  (-0.05, 0.16) |
| **Former heavy** | 0.03  (-0.07, 0.12) | 0.004  (-0.10, 0.11) | 0.01  (-0.11, 0.14) | -0.01  (-0.11, 0.08) | 0.05  (-0.05, 0.15) | 0.03  (-0.06, 0.11) | -0.05  (-0.18, 0.07) |
| **Current light** | -0.04  (-0.10, 0.02) | -0.09*  (-0.13, -0.02) | -0.07  (-0.13, 0.01) | -0.04  (-0.10, 0.01) | -0.02  (-0.07, 0.03) | 0.01  (-0.03, 0.06) | -0.04  (-0.10, 0.03) |
| **Current moderate** | -0.05  (-0.11, 0.02) | -0.06  (-0.13, 0.01) | -0.05  (-0.12, 0.03) | -0.03  (-0.09, 0.03) | -0.04  (-0.09, 0.01) | -0.01  (-0.07, 0.04) | -0.01  (-0.08, 0.06) |
| **Current heavy** | -0.06  (-0.16, 0.03) | -0.07  (-0.19, 0.05) | -0.01  (-0.12, 0.11) | -0.02  (-0.12, 0.07) | -0.07  (-0.14, 0.004) | -0.001  (-0.08, 0.08) | -0.18**  (-0.30, -0.07) |
|  | | | | | | | |
| Data presented as β (95% CI)  **Models** used the healthy cohort and were adjusted for: age, gender, race, SES, physical activity, smoking status, self-reported health, binge drinking, age^2^, age x gender, race x gender, race x SES, gender x SES. The healthy cohort excluded all individuals with a self-reported history of the following major chronic diseases: heart disease, cancer, peptic ulcer disease, cirrhosis, and chronic obstructive pulmonary disease.  **AL-** allostatic load  **SES** – socioeconomic status  *** p < 0.05; ** p < 0.01** | | | | | | | |

| **S1 Table C. Adjusted* association of AL with drinking categories by race** | | | | |
| --- | --- | --- | --- | --- |
|  | **β (95% CI)** | **P** | **β (95% CI)** | **P** |
| **Full MIDUS biomarker cohort** | **White**  **(n = 923)** |  | **Black**  **(n = 271)** |  |
| Lifelong abstainer / former light | reference |  | reference |  |
| Former moderate | -0.20 (-0.46, 0.07) | 0.14 | 0.07 (-042, 0.57) | 0.77 |
| Former heavy | -0.13 (-0.43, 0.17) | 0.40 | 0.01 (-0.56, 0.59) | 0.96 |
| Current light | -0.33 (-0.51, -0.14) | < 0.001 | 0.01 (-0.35, 0.36) | 0.97 |
| Current moderate | -0.33 (-0.52, -0.14) | < 0.001 | 0.07 (-0.34, 0.48) | 0.74 |
| Current heavy | -0.48 (-0.82, -0.13) | < 0.01 | 0.11 (-0.49, 0.70) | 0.73 |
| **Sensitivity analysis in healthy cohort**** | **White**  **(n = 646)** |  | **Black**  **(n = 200)** |  |
| Lifelong abstainer / former light | reference |  | reference |  |
| Former moderate | -0.35 (-0.65, 0.05) | 0.02 | 0.32 (-0.25, 0.89) | 0.27 |
| Former heavy | -0.05 (-0.41, 0.32) | 0.81 | 0.54 (-0.22, 1.30) | 0.17 |
| Current light | -0.39 (-0.60, -0.18) | < 0.001 | 0.02 (-0.38, 0.42) | 0.92 |
| Current moderate | -0.43 (-0.65, -0.20) | < 0.001 | 0.25 (-0.21, 0.72) | 0.29 |
| Current heavy | -0.59 (-1.02, -0.16) | < 0.01 | 0.14 (-0.53, 0.80) | 0.68 |
| *Adjusted for: age, gender, physical activity, smoking status, self-reported health, binge drinking, age^2^, age x gender, gender x SES, self-reported health x SES, as well as past medical history of heart disease, COPD, peptic ulcer disease, cirrhosis, cancer, and TIA/stroke.  ****Sensitivity analysis** cohort excluded all individuals with a self-reported history of the following major chronic diseases: heart disease, cancer, peptic ulcer disease, cirrhosis, and chronic obstructive pulmonary disease. Sensitivity analyses were adjusted for all covariates listed above except heart disease, cancer, peptic ulcer disease, cirrhosis, TIA/stroke and chronic obstructive pulmonary disease.  AL – allostatic load; SES – socioeconomic status; COPD – chronic obstructive pulmonary disease; TIA – transient ischemic attack | | | | |
